# Accidental carbon monoxide poisoning in our homes

**DOI:** 10.4103/0972-5229.58546

**Published:** 2009

**Authors:** Shruti Sharma, Rahul Gupta, Barinder S. Paul, Sandeep Puri, Shuchita Garg

**Affiliations:** **From:** Department of Critical Care, Dayanand Medical College and Hospital, Ludhiana, Punjab - 141 001, India; 1 Department of Medicine, Dayanand Medical College and Hospital, Ludhiana, Punjab - 141 001, India

**Keywords:** Carbon monoxide, carboxyhemoglobin, gas geyser, liquefied petroleum gas, magnetic resonance imaging

## Abstract

Carbon monoxide (CO) is a colorless, odorless, tasteless, nonirritating, but significantly toxic gas. It is a product of combustion of organic matter in presence of insufficient oxygen supply. Symptoms of mild poisoning include headaches, vertigo and flu like effects, whereas larger exposures can lead to significant toxicity of the central nervous system (CNS), heart, and even death. We are reporting two cases that presented to us in the winter months of December to January with history, sign, symptoms, and radiological evidence of suspected CO poisoning.

## Introduction

Carbon monoxide (CO) is one of the leading causes of accidental poisonings. Experts agree that it is difficult to estimate the incidence of CO poisoning cases, because the symptoms resemble many other common ailments.[1,2] This is more so in India, where there is improper reporting of morbidity and mortality attributable to suspected CO poisoning. High index of suspicion, clustering of such cases in winter months, and a careful history taking, help in making the diagnosis. Here, we are reporting two cases out of seven patients of suspected CO poisoning.

## Case Reports

### Case 1

A 11-year-old male child was admitted with a history of having been found unconscious in the bathroom by the elder brother of the patient. There was no evidence of associated tonic-clonic movements, tongue bite, frothing from mouth, vomiting, bladder, and bowel incontinence or trauma. There was no history of fever, seizures, headache, vomiting, and substance abuse prior to this episode. At the time of admission, patient was unresponsive and decerebrating. Pupils were normal size and well reacting to light, and plantar reflex was extensor bilaterally and systemic examination was normal. Patient was intubated, mechanically ventilated, and was started on antiedema measures and other supportive treatment. Magnetic resonance imaging (MRI) showed bilateral gyral swelling of the frontal and parietal lobes with reduced signal intensity in the bilateral caudate nuclei and putamen. Patient improved and was extubated on the fourth day of admission and was discharged in satisfactory condition after seven days without any neurological sequelae.

### Case 2

A 25-year-old male was found lying unconscious and brought out after breaking open the bathroom door after one hour. There was no evidence of seizures, vomiting, urinary and bowel incontinence or any kind of trauma at the time of incident and no history of fever, seizures, headache, and substance abuse in the past. Patient when brought was unresponsive. Pupils were 3.5 mm bilaterally reacting to light and plantar reflex was extensor on both sides. There was no neck rigidity, fever, and any cranial nerve deficit at the time of presentation. Patient's blood pressure was 90/50 mm Hg, which responded to fluid resuscitation. Systemic examination and investigations were normal. Level of consciouness improved over the next few hours, and he was managed with oxygen with a venturi mask, antiedema measures, and supportive treatment. MRI on the day of admission was normal. Patient's condition worsened on fourth day as his sensorium deteriorated and he developed flexor posturing. He was intubated and put on ventilatory support. Cerebrospinal fluid (CSF) examination was normal. MRI at this time showed diffuse gyral swelling with hyperintense signal in bilateral basal ganglia and focal areas of diffuse restriction in bilateral frontoparietal cortex. Patient developed fever and chest radiograph showed opacities on the right side. He was managed on the lines of ventilator-associated pneumonia with antibiotics and was extubated on the tenth day of admission. Patient was discharged after 25 days of hospital stay. He had residual hypoxic damage in the form of persistent vegetative state and was discharged to a domiciliary care on request.

## Discussion

Gas geysers have emerged as a cost-effective efficient method for heating water at homes. Gas geysers run on LPG, the combustion of which leads to generation of CO, hydrocarbons, and nitrogen oxides. These by-products when enclosed in a small space can result in serious morbidity and mortality in the victims. Sources of CO poisoning could be house fires, faulty furnaces, gas water heaters, wood burning stoves, motor vehicle exhaust, and various propane fuelled equipments, etc.[1,2]

CO toxicity occurs by competitive binding of CO to the hemoglobin heme groups with a resulting increase in the affinity of the remaining for oxygen, shifting the oxygen-hemoglobin dissociation curve to the left.[3] CO binds to cardiac and skeletal myoglobin also. A “rebound effect” with delayed return of symptoms may be due to late release of CO from myoglobin with subsequent binding to hemoglobin.[4]

The mean half-life of COHb is 320 minutes on room air, 80.3 minutes at 100% oxygen at one atmosphere, and 23.3 minutes at three atmospheres. Continued exposure to CO can lead to flu-like symptoms, headaches, dizziness, tiredness, and nausea that may progress to confusion, irritability, and impaired judgment, memory impairment and incoordination or even death. Normal COHb levels are less than 5%, up to 9% in cigarette smokers. Serious toxicity is associated with levels above 25%, and risk of fatality at 70%. In late presenting patients, a normal COHb level cannot be used to rule out poisoning.[5]

The predilection for the globus pallidus may relate to hypotensive effect of CO poisoning in the watershed territory of the arterial supply. Lesions within deep white matter have been reported as being indicator of poor prognosis compared to those in the globus pallidus.[6]

Treatment includes immediate removal of the victim from the exposure and administration of high-flow or 100% oxygen by a nonrebreather reservoir oxygen mask.[7] Hyperbaric oxygen (HBO) is also used in the treatment of poisoning, but consensus has still not been reached for or against its use.[8,9]

In case of our patients, diagnosis was made on the basis of history, response to supportive treatment, exclusion of other causes, and MRI evidence. Six of seven patients had no neurological sequelae of the illness. One patient (Case 2) had a prolonged course of disease and was discharged after a prolonged stay in the hospital. This patient also had residual neurological damage. The cause could be prolonged exposure to toxic levels of CO resulting in hypoxic brain damage.

Prevention always takes precedence over everything else. The geyser should not be switched on after locking the door from inside, ventilation should be kept open and gap should be maintained between two people taking bath to avoid increase in the CO density. Gas geyser unit should be placed outside the bathroom with a hose of hot water going inside. Gas geyser switch should ideally be at such a height that it can be switched off easily.[10]

Following these precautions can decrease the incidence, mortality, and morbidity due to accidental CO poisoning.
